# Reconstruction of Complex Cranial and Orbit Fractures with Associated Hemorrhages: Case Report and Review of the Literature

**DOI:** 10.7759/cureus.7694

**Published:** 2020-04-16

**Authors:** Andrew Caras, Christopher Alexander, Alexander Young, William Miller, Azedine Medhkour

**Affiliations:** 1 Neurological Surgery, The University of Toledo Medical Center, Toledo, USA; 2 Neurological Surgery, The University of Toledo College of Medicine and Life Sciences, Toledo, USA

**Keywords:** cranial reconstruction, traumatic optic neuropathy, craniofacial trauma

## Abstract

We present our experience following a unique case of coincident intracranial hemorrhage and comminuted fractures of both the squamous temporal bone and zygomaticofrontal orbit. Surgical techniques and outcome for this presentation have yet to be sufficiently described. A 55-year-old male presented following trauma with Glasgow Coma Scale score of 7. Radiographic evaluation revealed comminuted fractures of the squamous temporal bone with extension into the lateral orbit, along with zygomatic process fracture extending 2.5 cm medially into the orbital roof. Zygomaticofrontal orbital roof fragments reached superiorly into the middle cranial fossa and inferiorly into the orbit. Surgical intervention was deemed necessary to address underlying epidural hematoma, subarachnoid hemorrhage, correction of cranial bone defects, and decompression of the optic nerve and other intraorbital nerves. A frontotemporal approach was employed. Repair of temporal and orbital fractures was accomplished using a combination of wire mesh screws and titanium miniplates. Postoperative imaging demonstrated bony approximation and successful evacuation of traumatic hemorrhage. The patient remains functionally and neurologically intact apart from a sluggishly responsive left eye presumed to result from a left optic nerve or ciliary ganglion lesion. Although rapid reconstruction of complex cranial-orbital trauma and hematoma evacuation can permit acceptable gross functional neurological outcome following massive trauma, orbital fracture and subsequent hemorrhagic processes may be the nidus of neurological sequelae in this complex traumatic constellation. Thus, alterations in surgical approach and reconstruction are appropriate in order to maximize neurological function while supporting restoration of cosmetic space.

## Introduction

Operative planning and management of comminuted and depressed cranial fractures is complex, varying by fracture and location. Surgery is often required for compound or depressed fractures significant enough to damage underlying tissue, and when intracranial hematoma or hemorrhage is present [1,2]. Inherent to surgical management of cranial trauma is management of associated intracranial hemorrhage (ICH) with subsequent establishment of hemostasis. Temporal bone injuries occur in 4.7% of all skull fractures, while zygoma injuries occur in 17% of head trauma cases [3,4]. Orbital wall fractures, especially in concert with ICH and other cranial fractures, are among the most complex to reconstruct. Operative strategies are dictated by the portion of orbit involved, with the coronal approach often maximizing exposure of zygomaticofrontal, zygomaticosphenoidal, and zygomaticotemporal regions [5-7]. Maximal access to the fracture is vital for open reduction and stabilization with fixation, especially in the context of concomitant ICH [5]. 

We present a complex case following trauma associated with concomitant comminuted squamous temporal fracture, zygomaticofrontal orbital fracture, epidural hematoma (EDH), subarachnoid hemorrhage (SAH), and intraparenchymal hemorrhage (IPH). This trauma was successfully managed using a single frontotemporal question-mark style incision to achieve adequate intracranial exposure, fracture reconstruction, and hematoma evacuation. In this case report, we aim to catalog operative planning, management, and outcome of this complex traumatic presentation with emphasis on optic nerve (ON) functionality. We also present preoperative and postoperative radiography with intraoperative photos, cataloging the reconstructive process as an educational tool to better appreciate anatomical relationships. To date, there is a paucity of similar or related reports within the neurosurgical literature. 

## Case presentation

A 55-year-old male was transported by local emergency medical services as a level 1 trauma to the emergency department with severe injuries to the left temporal and orbital regions sustained from an assault. Glasgow Coma Scale (GCS) at presentation was 7 (E = 1, V = 2, M = 4) [8]. Large subdermal hematomas were observed in the left supraorbital, frontal, and temporal bone regions. Blood was seeping from both external nares and a 1 cm laceration on the left cheek. A posterior left rib fracture was also noted. Medical history was unobtainable. Initial neurological examination revealed an inappropriately dilated (4 mm) left pupil which reacted sluggishly to light compared to the right pupil (2 mm). Blink, corneal, and cough reflexes were intact. All four extremities withdrew from noxious stimuli. Further neurological assessment was prohibited by the patient being obtunded. The patient was sedated, intubated, and admitted to the neurointensive care unit. 

Preoperative radiography

**Figure 1:**
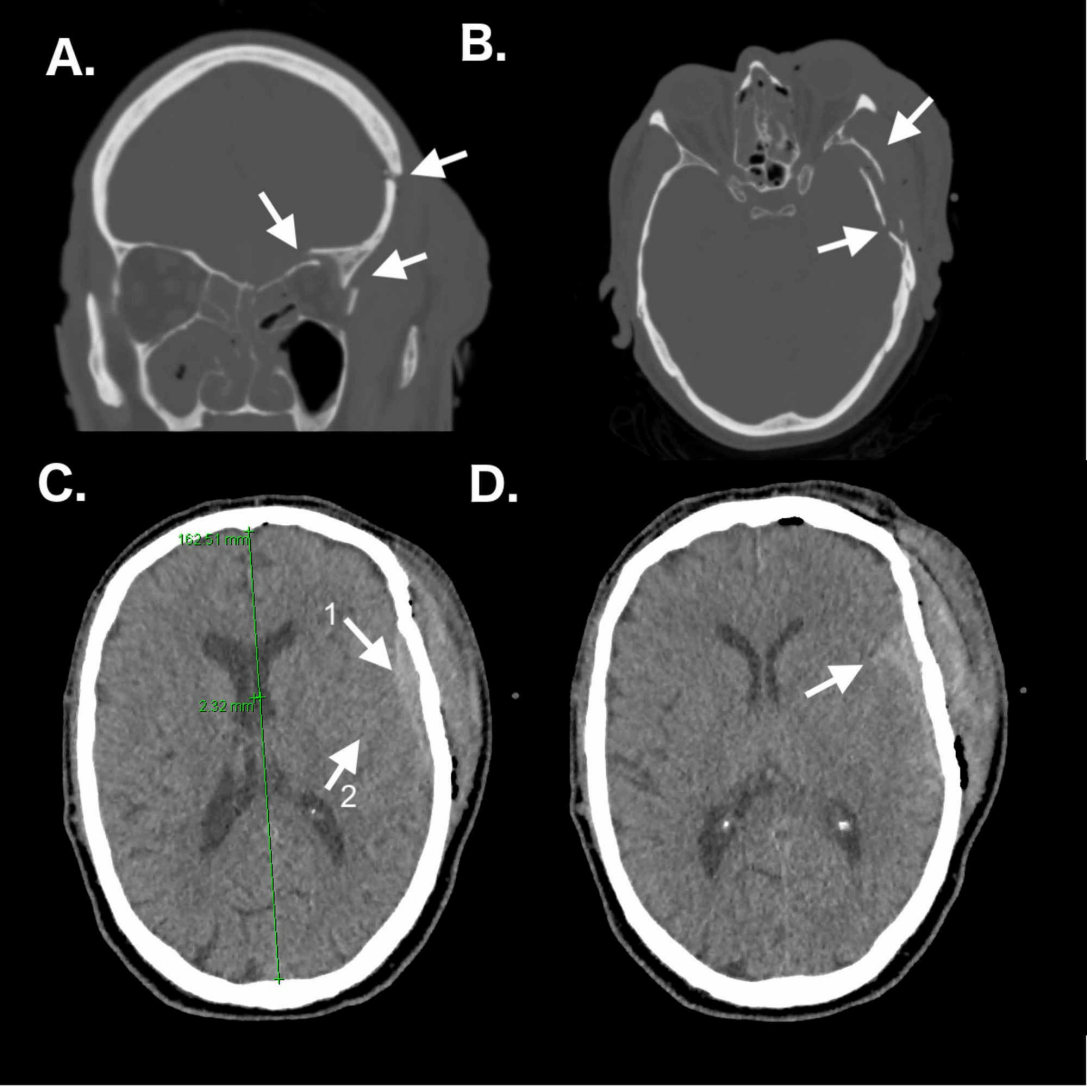
Preoperative non-contrast CT. (A) Coronal CT (bone window) showing comminuted fractures of the orbital roof, lateral orbital wall, and medial orbital wall. (B) Axial CT (bone window) demonstrating a depressed and comminuted fracture of the squamous temporal bone displaced medially with subcutaneous edema. (C) Axial CT (brain window) revealing a 7-mm epidural hematoma underlying the depressed squamous temporal fracture (arrow 1) with associated intraparenchymal hemorrhage and local cerebral edema (arrow 2), and approximately 2-mm midline shift as illustrated. (D) Axial CT (brain window) indicating subarachnoid hemorrhage with sulci effacement along the left insula, inferior to the epidural hematoma.

**Figure 2:**
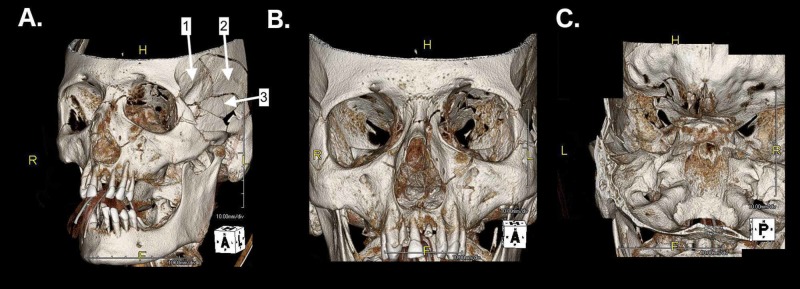
Three-dimensional CT radiography. (A) Anterolateral view illustrating multiple squamous temporal, zygomatic, and nasal fractures. Three major squamous temporal fracture components are labeled numerically. (B) Comminuted left orbital roof and lateral orbit fractures, as compared to the right orbit, with patency of the superior and inferior orbital fissures. (C) Intracranial view of the skull base. Multiple fractures in the left orbit are observed.

Initial non-contrast CT of the brain (Figure 1) revealed several cranial and orbital fractures and hemorrhagic processes. Three-dimensional CT reconstruction (Figure 2) was also performed to better appreciate the complex constellation of fractures. Specifically, depressed and comminuted fractures of the squamous portion of the left temporal bone were appreciated to extend into the left lateral orbital wall, left orbital roof, and the anterior left zygomatic bone. Comminuted fractures of both the zygomatic process and the orbital roof were observed, with bony protrusions into the posterior orbit and calvarium. Cranial bone fragments were found both in the middle fossa and the orbit. Stranc lateral oblique type left nasal bone fracture with depression of bone and a mildly and inferiorly displaced (1-2 mm) fracture near the midline of the left sphenoid sinus roof were noted [9]. Strikingly, no optic sheath hematoma or ON bony impingement was observed. CT also revealed several left frontotemporal hemorrhagic processes, including 7 mm EDH with 2-mm midline shift, SAH including the anterior and middle fossae, and IPH suggested by foci of increased attenuation. High attenuation of the paranasal sinuses suggested hemorrhage, likely originating from zygomaticofrontal fracture. Cervical spine CT was unremarkable.

Operative technique

Operative planning targeted elevation and resection of depressed left temporal bone fractures, ICH evacuation, exploration of the subarachnoid space, reconstruction of the orbit, and re-approximation and reconstruction of temporal fractures. Surgery began within 12 hours of patient presentation. The patient was placed in a supine position with a left shoulder roll and the head on a cerebellar headrest. A left question-mark style frontal-temporal-parietal incision extending from the medial frontal hairline, extending posteriorly over the parietal bone, and terminating anterior to the tragus was chosen to maximize exposure. Once the temporalis muscle and fascia were elevated to expose the preauricular and frontal portions of the zygomatic bone, three large and several small fragments of the comminuted temporal bone were seen. 

**Figure 3:**
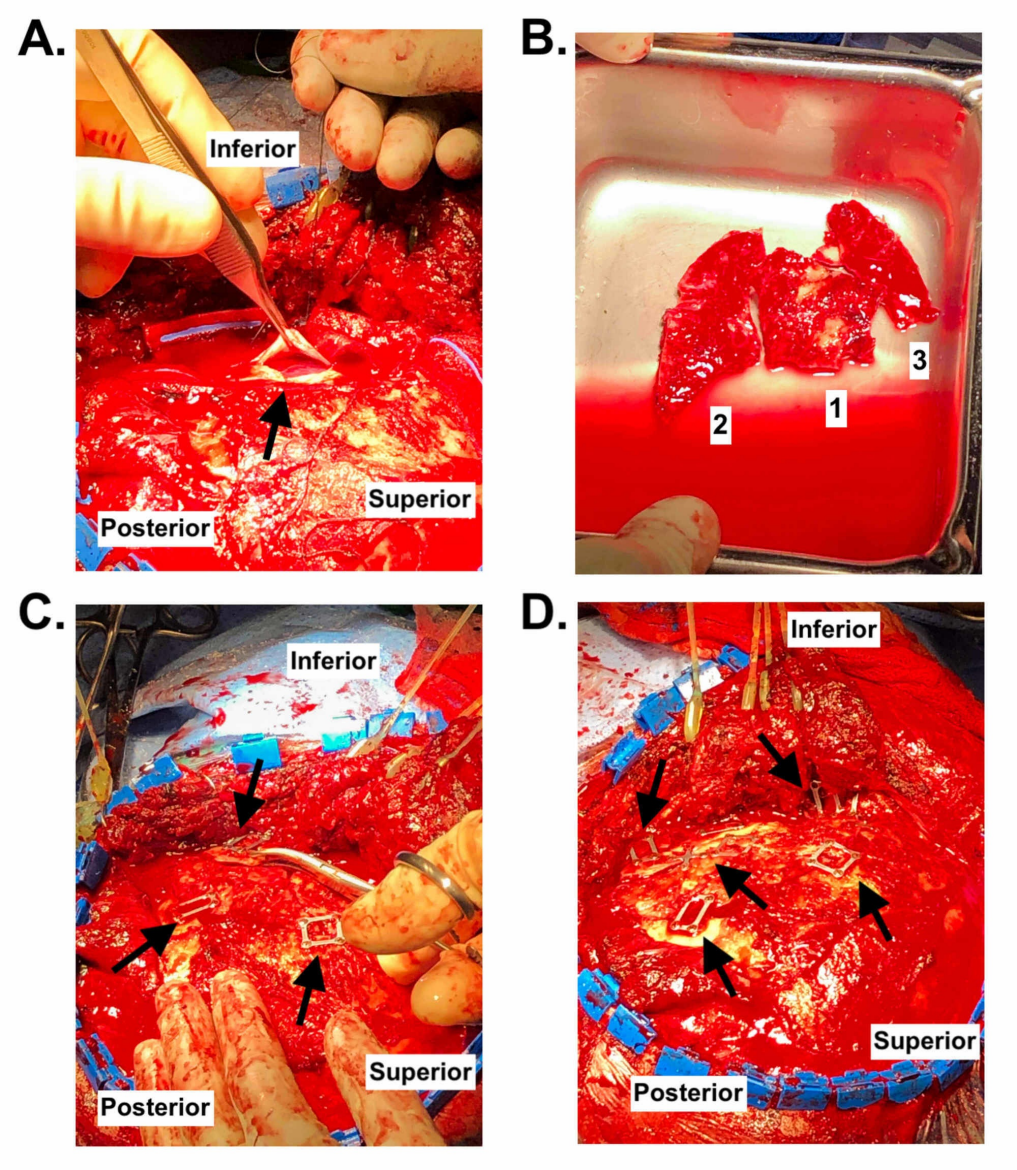
Intraoperative photos of the cranial reconstruction. (A) Dura opening demonstrating lack of subdural hematoma. (B) Temporal bone fragments before fixation, numerically labeled identically to Figure 2A. (C) Repositioning of orbital bone fragments. Location of two miniplates is also shown. (D) Reconstruction of comminuted fractures using miniplates. Orbital reconstruction is shown along the anterior edge of the operative field, with temporal reconstruction in the middle of the field.

Management of the temporal fracture began by drilling the edge of the largest component of the temporal fracture (#2 as seen in Figure 3B) to permit its elevation. Further dissection allowed the lateral orbital bone protruding into the orbit to be retrieved and resected, followed by the smaller fragments. Three large fragments of temporal bone were resected and irrigated. EDH was evacuated, and further hemostasis was achieved. Dural incision at the middle temporal region was performed to rule out the presence of subdural hematoma. Minimal quantity of blood-tinged cerebrospinal fluid (CSF) was suctioned briefly to clear the SAH before suturing the dura closed in the usual water-tight fashion.

Cranial reconstruction initially centered on approximation of the larger bone fragments with subsequent incorporation of smaller fragments to best re-establish normal anatomical topography. Reconstruction was planned from deep to superficial: first addressing the orbital fractures, and then reconstructing the large temporal fractures, followed by replacement of the smaller temporal fracture components. The previously retrieved and irrigated orbital wall fragments were replaced, approximated and attached using wire mesh screws. Large previously resected temporal fragments were then similarly irrigated, attached to adjacent bone fragments, and attached to surrounding intact bone using microplates and screws. Intact temporal bone was elevated with a Penfield 4 (Novo Surgical Inc, Oak Brook, IL) to re-establish normal anatomical orientation while adjacent smaller bone fragments were screwed into place piecewise. Smaller components of temporal bone reconstruction were performed in a stepwise fashion with surrounding anatomical structures being kept in view, preserving normal contour and re-establishing structure integrity. Intraoperative photos detailing key steps of this case are available in Figure 3. A Jackson-Pratt drain was inserted. Postoperatively, the patient remained intubated and was taken to the neurointensive care unit. 

Postoperative course

The patient was extubated and weaned from sedation with appropriate pain control on postoperative day (POD) 1, with a GCS of 13 (E4, V4, M5) one hour later. Upon relinquishing sedation, the patient’s Riker Sedation-Agitation scale score was 7 but quickly improved to 4 [10]. The Jackson-Pratt drain was removed on POD 2. On POD 3, speech was not clear, but the patient could state his name and follow commands with GCS of 14 (E4, V4, M6). Ophthalmic examination on POD 7 revealed a persistently dilated left pupil but fundoscopic examination of the left eye was unremarkable. The patient was stepped down from the neurointensive care unit to the general surgical floor on POD 8. Neurological examination on POD 15 revealed the left pupil was still inappropriately dilated (5 mm) and sluggishly reactive to light compared to the left (3 mm). Extraocular movements were intact bilaterally. Facial movements were symmetric, and the tongue protruded at midline. No ataxia was noted on finger-to-nose testing. The speech remained mildly dysarthric but improved to fluency on POD 16, at which time the patient was oriented to person, place, and time. Cranial incision sutures were removed on POD 18 without drainage, tenderness, or warmth. The patient was discharged on POD 29 to an extended care facility to undergo physical rehabilitation. 

At follow-up seven weeks postoperatively, the patient complained of moderate neck pain and new-onset mild short-term memory loss. The left pupil remained fixed at 4 mm and unreactive to light. The patient described being only able to see shapes in the left eye, likely due to ON injury from the trauma. Surgical incision was well approximated without edema, erythema, or drainage, and the skull appeared to have normal contours. The remainder of the exam was unremarkable, and the patient was counseled to follow up at his discretion.

Postoperative radiography 

**Figure 4:**
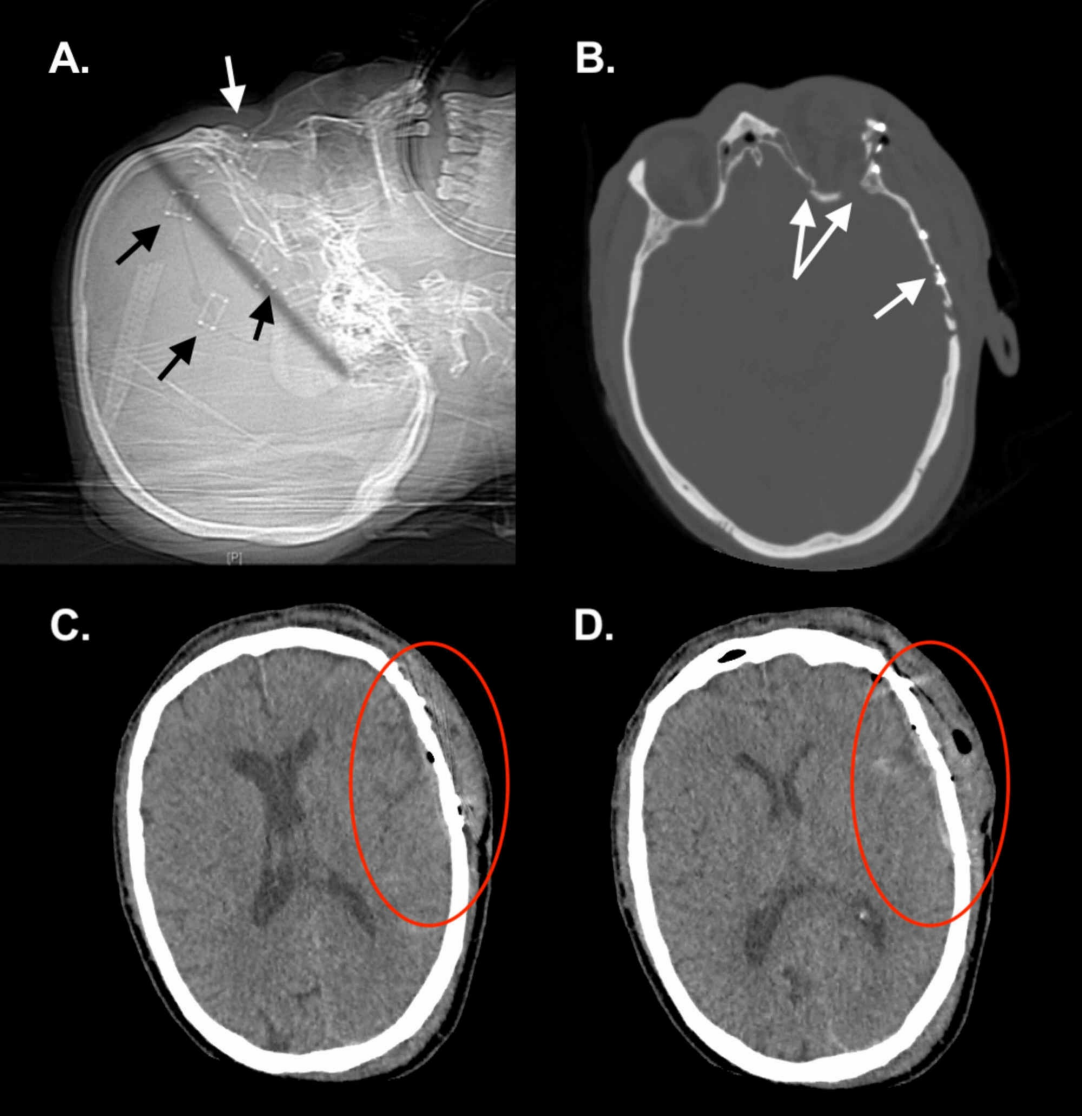
Postoperative radiography at 18 hours (non-contrast CT) and 18 days (MRI). (A) Sagittal scout CT with placement of temporal (black arrows) and orbital (white arrow) miniplates. (B) Reduction of comminuted temporal and lateral orbital wall fractures (bone window). A medial and posterior orbit fracture is still present. (C) Cleared subarachnoid hemorrhage (red oval; brain window) with minimal pneumocephalus appreciated. (D) Evacuated epidural hematoma (red oval; brain window).

Radiologically, non-contrast head CT performed 18 hours postoperatively (Figure 4) demonstrated successful hematoma evacuation and temporal bone reconstruction.

## Discussion

This case highlights that unconventional cases may require several techniques to achieve an acceptable outcome. There is a paucity of literature that describes the presentation, surgical management, and outcome following complex and combined squamous, zygomatic, and orbital fractures with concurrent ICH. Indications for surgical intervention for cranial fractures are based upon either the fracture characteristics or the presence of intracranial bleeding [1,11]. Characteristics requiring surgical intervention include compound fracture, a depression thick enough to damage underlying tissue, and the presence of significant intracranial hematoma or hemorrhage [1,2]. Bullock et al. recommended assessing EDH size, GCS at presentation, and pupillary abnormalities to determine when surgery is appropriate, and established that GCS less than 9 and anisocoria associated with EDH at presentation are indications for immediate surgical intervention [11]. The case herein presented with GCS 8 and enlarged pupil on the side of injury, which alongside the other numerous traumatic factors supporting surgical intervention, validated the recommendations by Bullock et al.

When evaluating the surgical approach used, independent orbital pathology causing ON deficits can be effectively managed with supraorbital and transorbital approaches [12]. Similarly, Dye et al. described evacuation of spontaneous SAH using a supraorbital approach [13]. However, combined orbital and temporal fractures in the setting of multiple ICH called for a large, single question-mark style incision to expose the extent of both fractures and permit exposure and evacuation of ICH. Yang et al. recently discussed how a smaller, “n-shape” incision may permit at least equal utility for decompressive craniectomy [14]. Potential benefits of novel approaches for reconstructive craniotomy of the temporal-orbital area, especially in the setting of complex fractures, require future investigation.

Temporal fractures are typically divided into two categories relative to the petrous ridge: transverse or longitudinal [15]. However, most cases, including the case herein, cannot be clearly delineated in this manner. Diaz et al. described that separating fractures into these two categories is not prognostic for neurological deficits, and argued for segregation into “otic capsule sparing” and “otic capsule disrupting” cohorts [15]. Otic capsule disrupting fractures are highly associated with sensorineural hearing loss, facial nerve paralysis, nerve disruption, CSF leaks and fistulae, and intracranial complications. Otic capsule sparing fractures, although generally associated with conductive or mixed hearing loss, impart a better neurological outcome [15]. Temporal fracture in our case spared the otic capsule, resulting in no hearing loss or facial palsy, demonstrating that the presence of complex temporal trauma may not be the inciting event for neurological sequelae.

Neurologically, at the last follow-up our patient had an unreactive left pupil and left eye visual acuity deficits in the setting of intact extraocular movements. This is representative of an ON lesion. The presence of a ciliary ganglion lesion could also be considered in this case as the left pupil was unreactive to bilateral light; however, with the loss of visual acuity in the left eye, an ON lesion is more likely as a ciliary ganglion lesion would be unlikely to significantly affect both near and far visual acuity. We surmise these lesions were caused by the orbital roof fractures, as well as remnant hemorrhagic material in the medial orbit. Although orbital reconstruction was grossly achieved within 12 hours of injury, and full deep orbit reconstruction was not performed (as seen in Figure 4B) in an attempt to minimize iatrogenic nerve deficits, the patient did not return to full ON functionality in the left eye. Tan et al. recently published a similarly complex case; however, the patient regained full ON function which may be due to evacuation of a bony spur causing ON impingement [16]. Recommendations for orbital reconstruction following trauma are controversial, with this case adding to the controversy [17]. Recent criteria for orbital fracture reconstruction do not specify timing indications for cases such as the one described herein [18]. Rapid orbital repair with assurance of nearby nerve decompression, alongside hemostasis and alleviation of subsequent mass effect, may be the chief goal of surgical intervention in these complex traumatic scenarios. This is suggested by recent findings in the literature and should be further investigated [19]. We additionally propose that in similar cases with combined temporal and orbital trauma with multiple ICH foci, the orbital fracture may be the nidus of neurological sequelae even without obvious preoperative nerve compression. Causes of functional deficits resulting from similarly complex traumatic cases should be studied alongside the role of time to operation, age, mechanism of injury, surgical approach, and other coincident factors. Future study assessing the role of orbital reconstruction to restore ON functionality in similarly complex traumatic cases may help elucidate its role.

Concurrent treatment of this scenario of cranial fractures with multiple origins of intracranial bleeding was challenging, necessary, and unique. For these reasons, extensive photographic and radiologic documentation of this case was undertaken. We intend for this complicated case to demonstrate techniques to manage multiple fractures and hemorrhages and follow the postoperative neurological sequelae of such phenomena. However, this study is limited by both its low power as a single case report to demonstrate effectiveness of the surgical methodology herein and the relatively short follow-up period. Thus, we find it cogent to present our findings as an educational tool to improve knowledge of both radiology-anatomy correlations and operative planning in the setting of complex neurosurgical trauma. We additionally aim to motivate study of similarly complex traumatic cases to classify ideal approaches, elucidate time-to-operation recommendations to maximize outcome, delineate the role of orbital reconstruction and nerve decompression, and characterize resulting neurological deficits.

## Conclusions

Presented herein was a unique case of multiple comminuted cranial and facial fractures alongside multifocal ICH. Rapid clinical and radiographic assessment permitted full appreciation of the extent of fractures extending from the squamous temporal bone into the orbital roof and zygomaticofrontal bone, as well as the underlying bleeding. This information guided the scope and direction of the indicated surgical intervention and incision. Following exploration and repair of ICH, bone fragments were repositioned and fixated employing a stepwise reconstruction to minimize neurological sequelae and maintain the cranial contour. Ultimately, the patient remains with a presumed left ON lesion but is otherwise functionally intact. In this case, we emphasize that rapid reconstruction following massive cranial trauma in the presence of multiple ICHs can be effectively managed with good gross neurological outcome. The role and specific characteristics of orbital reconstruction to minimize focal neurological deficits in similarly complex trauma remain to be elucidated.
